# Supplementary figures and images for: Ultrasonographic Diagnosis and Computed Tomographic Confirmation of a Scapular Body Stress Fracture in an Elite Boxer: A Case Report
Source: Diagnostics (Basel). 2025 Oct 11;15(20):2565. doi: 10.3390/diagnostics15202565 (PMC12563319; doi:10.3390/diagnostics15202565)

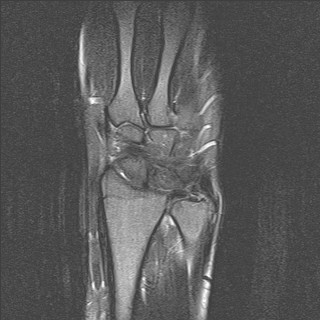

Supplement: Supplementary file 1 [file diagnostics-15-02565-s001.zip › Figure S1.jpg]

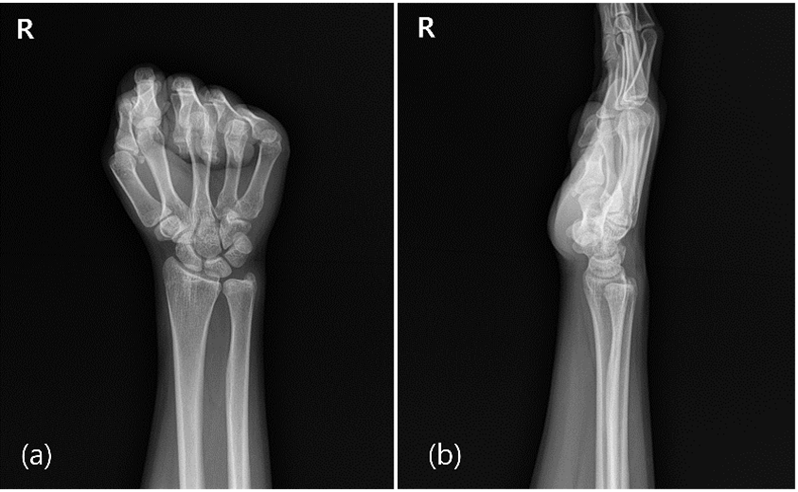

Supplement: Supplementary file 1 [file diagnostics-15-02565-s001.zip › Figure S2.png]
